# Two new species of *Solanum* (Solanaceae) from the Amotape-Huancabamba Zone of southern Ecuador and northern Peru

**DOI:** 10.3897/phytokeys.1.660

**Published:** 2010-11-01

**Authors:** Stephen Stern, Lynn Bohs

**Affiliations:** Department of Biology, University of Utah, 257 S 1400 E, Salt Lake City, Utah 84112, U.S.A.

**Keywords:** Amotape-Huancabamba Zone, Andes, Ecuador, Huancabamba Depression, new species, Peru, *Solanum*

## Abstract

Two new species of Solanum subgenus Leptostemonum from southern Ecuador and northern Peru are described here. Solanum rubicaule S. Stern, **sp. nov.**, is a member of sect. Torva and is characterized by a festooning, scandent growth form and fruits held horizontally on recurved pedicels. Solanum achorum S. Stern, **sp. nov.**, is a member of sect. Erythrotrichum and is characterized by 4–12-flowered inflorescences, small seeds, and a small calyx. Both species are distributed in the Amotape-Huancabamba Zone of the Andes in northern Peru and southern Ecuador.

## Abstract (sp)

Se describen aquí dos especies nuevas de Solanum subgenero Leptostemonum del sur de Ecuador y el norte de Peru. Solanum rubicaule S. Stern, **sp. nov.**, es un miembro de la sección Torva y se la caracteriza por su hábito escandente y frutos sostenidos laterales por pedicelos recurvados. Solanum achorum S. Stern, **sp. nov.**, es un miembro de la sección Erythrotrichum y se la distingue por infloresencias con pocas flores, semillas pequeñas, y un cálice pequeño. Ambas especies ocurren en la zona Amotape-Huancabamba en los Andes del norte de Peru y el sur de Ecuador.

## Introduction

The Amotape-Huancabamba Zone of northern Peru and adjacent southern Ecuador, also known as the Huancabamba Depression due to the low summits of the Andes in this area, is one of the most biodiverse regions of the neotropics (Berry 1982, Ayers 1999, Weigend 2002, 2004, Stern et al. 2008). Its diversity can be attributed to a number of factors including the heterogeneity of its topography, vegetation, geological substrate, and climate. The complex topography, resulting from the Andes dissected by the Huancabamba, Marañon, and Utcubamba rivers, is likely the most important factor influencing its biodiversity, with the mountains and rivers creating a mosaic of isolated valleys and habitat fragments that promotes speciation (Panero 1992, Weigend 2002). This region is a hotspot of endemism for selected groups of Solanum (Knapp 2002a). Recent collecting trips to this area have yielded a vast number of Solanum specimens including the two species described here.

Both of these new species belong to Solanum subgenus Leptostemonum (Dunal) Bitter, a group characterized by long, attenuate anthers, stellate hairs, and epidermal prickles. An extensive phylogenetic study of this group resolved subgenus Leptostemonum as monophyletic and delimits 12 to 15 major subclades (Levin et al. 2006, Weese and Bohs 2007). The two species described here are both difficult to place within the subgenus using morphological features because they share characteristics with multiple groups. Therefore, DNA sequences were obtained from these two species and analyzed using the methods and framework phylogeny of Levin et al. (2006) to confirm their relationship to other members of Solanum subgenus Leptostemonum. One of the species described here, Solanum rubicaule S. Stern, has morphological and molecular characters that place it in Solanum section Torva Nees. This group contains approximately 40–50 species of erect or scandent shrubs or small trees typically with branched inflorescences, straight or recurved spines, and triangular corolla lobes with abundant tissue between the petals (interpetalar tissue).

The second new species, Solanum achorum S. Stern, belongs to Solanum section Erythrotrichum Child. This group has approximately 23 species and is characterized by plurifoliate sympodial units (see Knapp 2002b for description of sympodial units in Solanum), recurved prickles, a ferruginous to reddish tomentum with stellate-glandular hairs, and large berries with a pubescent exocarp (Agra 2008).

Morphological and molecular work has revealed Solanum rubicaule and Solanum achorum to be distinct species within in their respective clades. (S. Stern and L. Bohs, unpubl. data). 

## Taxonomic treatments

### Solanum section Torva

#### Solanum rubicaule

S. Stern sp. nov.

urn:lsid:ipni.org:names:77107690-1

Figs 1 2 

##### Latin

Solano subinermi *Jacq. et* S. asperolanato *Ruiz & Pav. similis sed a* S. subinermi *pedicellis fructiferis curvatis, a* S. asperolanato *habitu scandenti differt.*

##### Type.

**Peru:** Cajamarca: Prov. San Ignacio, road from San Ignacio to El Chaupe, 2–3 km hike in from trailhead to El Chaupe, 5°11'56"S, 79°03'51"W, 1775 m, 17 December 2007 (fl, fr), S. Stern et al. 181 (holotype: USM!; isotypes: BM001016784!, HAO [destroyed], NY00986627!, NY00986637!, UT!).

##### Description.

Scandent shrub, often festooning over other plants, 1–3 m tall. Stems armed with recurved, tan to orange roselike prickles to 3 mm in length, the base 2–3 × 0.5–1 mm, moderately to densely pubescent with tan to rusty, porrect-stellate hairs, the stalks 0.5–1 mm, multiseriate, the rays 5–10, 0.1–0.2 mm, unicellular to multicellular, the midpoints nearly absent, the lateral rays often partially proximally fused (see Roe 1971 for hair terminology). Flowering portions of stem consisting of difoliate sympodial units, the leaves usually geminate, those of a pair often slightly unequal. Leaves simple, the blades 10–13 × 5–8 cm, elliptic to ovate, chartaceous to coriaceous, discolorous, adaxially reddish brown, abaxially whitish green, the adaxial surface densely pubescent with multicellular, uniseriate glandular hairs 0.3–0.6 mm long, and stellate hairs like those of the stem but with the stalks ca. 0.2–0.6 mm, the rays 3–8, ca. 0.2–0.4 mm long, the abaxial surface densely stellate-pubescent with hairs like those of the stem but with the stalks 0.1–0.3 mm, the rays 8–12, ca. 0.2–0.4 mm long; venation pinnate, the secondary veins 5–7 on both sides of the midvein, the midrib abaxially occasionally with a few recurved spines like those of the stem; base obtuse, often asymmetrical; margin entire; apex acute; leaves subsessile to shortly petiolate (to 2 cm), the petiole moderately to densely pubescent with hairs like those of the stem, occasionally sparsely armed with recurved spines like those of the stem. Inflorescences to 12 cm, extra-axillary or subopposite the leaves, unbranched to twice branched, with 2–8 flowers, the plants andromonoecious, with male flowers on young plants and hermaphroditic flowers on older plants, the axes moderately to densely pubescent with hairs like those of the stem, unarmed; peduncle 0.5–3 cm; rachis 2–8 mm; pedicels 7–11 mm in flower, 10–20 mm in fruit, distally swollen, spaced 2–4 mm apart, articulated at the base. Flowers 5-merous. Calyx 1.2–2 cm long, the tube 2–3 mm, the lobes 12–18 × 3–6 mm, triangular, densely pubescent abaxially with hairs like those of the stem; fruiting calyx slightly accrescent, incompletely covering the fruit. Corolla 3–4 cm in diameter, chartaceous, white, stellate with moderate interpetalar tissue, lobed nearly to the base, the lobes 12–16 × 4–8 mm, narrowly triangular-ovate, slightly reflexed at anthesis, densely pubescent abaxially on midvein with hairs like those of the abaxial leaf surface, adaxially glabrous. Stamens 8–12 mm; filaments 1–2 mm long, glabrous; anthers 7–10 × 1–2 mm, attenuate, connivent, yellow, linear-lanceolate, tapering, the base cordate, the apex acute, with pores directed slightly introrsely, not opening into longitudinal slits. Ovary moderately stellate-pubescent with white hairs like those of the stem; style in functionally male flowers 4–7 × 0.5–1.5 mm, not exserted beyond stamens, cylindrical, glabrous; style in hermaphroditic flowers 10–14 × 0.5–1.5 mm, exserted beyond stamens, cylindrical, glabrous; stigma to 1.5 mm wide, capitate. Fruit a berry, 1–2 cm in diameter, globose with a small acute protrusion at the apex, green, hard at maturity, glabrous, the pedicels recurved down in fruit positioning the fruits horizontal to the rachis. Seeds 25–50 per fruit, reniform, brown, rugose, ca. 3 × 2.5 mm, flattened, with a small notch where connected to placenta.

**Figure 1.**
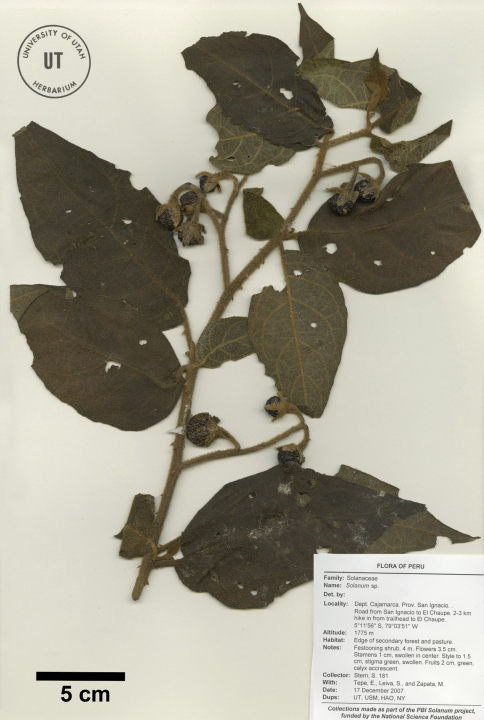
Isotype of Solanum rubicaule S. Stern [Stern et al. 181 (UT)].

**Figure 2.**
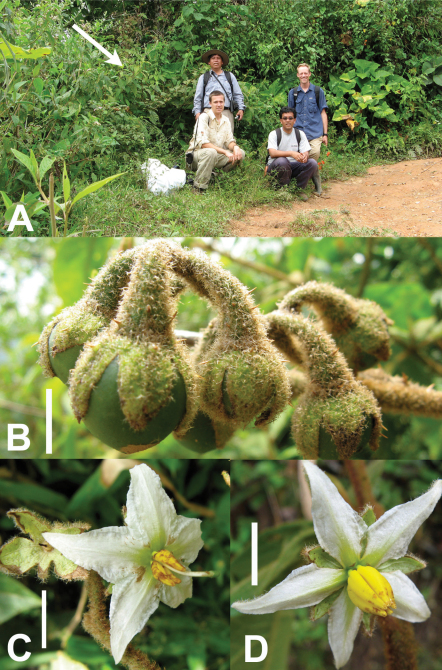
Photos of type collection of Solanum rubicaule S. Stern. **A** Collecting party in front of type collection, indicated by arrow, at trailside habitat in San Ignacio, Dept. Cajamarca, Peru (from left to right: Segundo Leiva, Stephen Stern, Mario Zapata, and Eric Tepe). **B** Fruiting inflorescence; note recurved pediels. **C** Hermaphroditic flower. **D** Functionally male flower; note the absence of exserted style. Scale bars = 1 cm.

##### Distribution.

Known only from northern Peru in Dept. Cajamarca and southern Ecuador in Prov. Zamora-Chinchipe in open places in disturbed montane tropical forest, 1650–2200 m in elevation.

##### Ecology.

The flowering specimen was collected in December. Fruiting specimens were collected in December–January and March–April.

##### Etymology.

The name Solanum rubicaule is derived from the festooning growth form, reminiscent of the genus Rubus L. and the Latin “*caulis*” for stem.

##### Conservation status.

According to the IUCN Red List Categories (IUCN 2010) Solanum rubicaule is classified as VU-B1a+B2a+B2biii; D2 (Vulnerable). The extent of occupancy is estimated to be approximately 10,000 km2 and less than five collected locations. This area of the Amotape-Huancabamba Zone has been underexplored, but collections have increased in recent years, largely due to efforts by MO in southern Ecuador and HAO in northern Peru. As this collecting continues and more specimens are determined in herbaria the number of locations should rise. Additionally, although there is continuing decline in forest habitat in this region due to deforestation for the establishment of settlements and farming, the effects of this on Solanum rubicaule are difficult to assess because it occurs in disturbed edges of forest and roadsides.

##### Specimens examined.

**Ecuador:** Zamora-Chinchipe: Cantón Chinchipe, Parroquía Zumba, trail from Guaramizal to cabin of Sandy León, W of Escuela Byron Jiménez, just S of Las Pircas, 4°46'60"S, 79°12'18"W, 2100 m, 28 March 2005 (fr), L.Bohs et al. 3336 (QCNE, UT); same locality, same date (fr) L.Bohs et al. 3338 (QCNE, LOJA, UT); same locality, 4°46'50"S, 79°12'33"W, 2000 m, 29 March 2005 (fr), L.Bohs et al. 3357 (QCNE, UT); Fundación Arco Iris, between Loja and Zamora, trail from field station to Río San Francisco, 3°59'20"S, 79°05'35"W, 2200 m, 5 April 2005 (fr), L.Bohs et al. 3425 (LOJA, QCNE, UT). **Peru:** Cajamarca: Prov. San Ignacio, above San Francisco (ca. a El Chaupe), 1650 m, 5 January 1995 (fr), S.Leiva et al. 1621 (HAO [destroyed], NY).

##### Discussion.

Solanum rubicaule has a festooning growth form, meaning that it is often arched and draping over other vegetation. This growth form is similar to members of Solanum sect. Micracantha Dunal, a group of vining species from the New World tropics that climb using recurved prickles. This superficial similarity explains why specimens of Solanum rubicaule are often annotated as “Solanum sect. Micracantha.” However, other morphological and molecular characters place Solanum rubicaule in Solanum sect. Torva, including flowers with triangular corolla lobes with abundant interpetalar tissue and typically branched inflorescences. Parsimony analyses of sequence data from three molecular markers (nuclear ITS and *waxy* or GBSSI and chloroplast *trnT-F*) also place Solanum rubicaule in sect. Torva; however, the relationships within the section are not well-resolved and require further study (S. Stern and L. Bohs, unpub. data).

Following the definition of Walker and Whelan (1991), the breeding system of Solanum rubicaule is andromonoecious, meaning that there are staminate and hermaphroditic flowers on the same plant. However, a more specific description of the breeding system might be “temporally andromonoecious” since the first-formed inflorescences on a plant appear to be composed entirely of male flowers. Inflorescences on older plants are composed of hermaphroditic flowers.

Within sect. Torva, Solanum rubicaule is similar to Solanum subinerme Jacq., a species found throughout northern South America from the Guianas to central Peru, both of which have a scandent growth form and few-branched inflorescences. However, Solanum rubicaule has a distinctive infructescence with fruits held horizontal to the rachis due to pedicels that curve downward (see Fig. 2b) while Solanum subinerme has fruits held upright on erect pedicels. The adaxial leaf surface of Solanum rubicaule is unarmed, while the adaxial leaf surface of Solanum subinerme often has straight prickles to 1.5 cm long. Both species have multiseriate stalked hairs on the adaxial leaf surface but those Solanum subinerme are nearly sessile to short stalked (to ca. 0.4 mm) and very thin (ca. 0.1 mm in diameter) while those of Solanum rubicaule reach 0.6 mm with greatly thickened stalks (to 0.3 mm in diameter). Herbarium specimens of Solanum rubicaule and Solanum asperolanatum Ruiz & Pav. are very similarwith regard to pubescence and flower appearance, but the latter species has upright inflorescences that are more than twice branched, typically has >12 flowers, is a large shrub or small tree and does not have the festooning growth form of Solanum rubicaule.

### Solanum section Erythrotrichum

The second new species, Solanum achorum, has morphological and molecular characters that place it in Solanum sect. Erythrotrichum (S. Stern and L. Bohs, in prep). Morphologically, this species shares the plurifoliate sympodial units, recurved prickles, ferruginous tomentum with stellate-glandular hairs, and berries 1.5–2.5 cm in diameter with a pubescent exocarp typical of other members of sect. Erythrotrichum. This group appears to have three distinct centers of diversity, in Central America, northeastern Brazil, and the Andes of Peru and Ecuador (Agra 2008).

#### Solanum achorum

S. Stern sp. nov.

urn:lsid:ipni.org:names:77107691-1

Figs 3 4 

##### Latin

Solano megaspermo *Agra et* S. velutino *Dunal affinis sed a* S. megaspermo *inflorescentiis paucifloribus et seminibus parvioris, a* S. velutino *calycibus parvioris differt*.

##### Type.

**Peru:** Amazonas: Prov. Chachapoyas, road from Leimebamba to Chachapoyas, about 15 km N of Leimebamba along Río Utcubamba, 6°37'39"S, 77°48'44"W, 2050 m, 13 December 2007 (fl, fr), S. Stern et al. 129 (holotype: USM!; isotypes: BM001016783!, HAO! [destroyed], NY00986767!, UT!).

##### Description.

Erect to scandent shrub, 1–3 m tall. Stems armed with recurved, tan to orange roselike prickles to 2.5 mm in length, the base 2–3 × 0.5–1 mm, sparsely to moderately pubescent with rusty, porrect-stellate hairs, the stalks nearly absent to 0.2 mm, multiseriate, the rays 5–10, 0.2–0.3 mm, unicellular to multicellular, the midpoints nearly absent. Flowering portions of stem consisting of plurifoliate sympodial units, the leaves apparently not geminate. Leaves simple, the blades 6–20 × 2.5–11 cm, elliptic to ovate, chartaceous, discolorous, adaxially dark green, abaxially whitish green, the adaxial surface sparsely stellate-pubescent with hairs like those of the stem, the abaxial surface moderately to densely stellate-pubescent with hairs like those of the stem but white and with midpoints often gland-tipped, these mixed with multicellular, uniseriate glandular hairs 0.3–0.6 mm long; venation pinnate, the secondary veins 4–5 on both sides of the midvein, the midrib abaxially occasionally with sparse recurved spines like those of the stem; base obtuse, often asymmetrical; margin entire; apex acute; petioles 0.5–3 cm, moderately pubescent with hairs like those of the stem, occasionally armed with sparse recurved spines. Inflorescences 2–15 (20) cm, 2–5-branched, with 4–12 flowers, the plants andromonoecious, specifically androgynoecious (Walker and Whelan 1991), with hermaphroditic flowers at the base of the inflorescence and occasionally with staminate flowers at the tip, the axes moderately to densely pubescent with hairs like those of the stem; peduncle 1–3 cm; rachis 3–5 cm; pedicels 4–15 mm in flower, 10–25 mm in fruit, erect, spaced 3–5 mm apart, articulated at the base. Calyx 3–6 mm long, the tube 1–3 mm, the lobes 2–4 × 1–2.5 mm, triangular, moderately to densely pubescent with hairs like those of the stem; fruiting calyx not accrescent, not completely covering the fruit. Corolla 2–3.5 cm in diameter, stellate with little to no interpetalar tissue, chartaceous to membranaceous, white, the tube 2–4 mm long, the lobes 10–15 × 3–5 mm, narrowly triangular-ovate, often reflexed, acute at apices, densely pubescent abaxially with white stellate hairs like those of the stem, glabrous to sparsely pubescent adaxially. Stamens 6–10 mm; filaments to 1 mm long, glabrous; anthers 6–10 mm × 1–2 mm, attenuate, connivent, yellow, the base cordate to obtuse, the apex acute to obtuse, the pores apical, not opening into slits. Ovary moderately pubescent with stellate hairs; style in functionally male flowers 3–6 × 0.5–1.5 mm, not exserted beyond stamens; style in hermaphroditic flowers 8–12 × 0.5–1.5 mm, exerted beyond stamens, cylindrical, moderately pubescent with stellate hairs; stigma 1.5–2 mm wide, capitate. Fruit a berry, 1.5–2 cm in diameter, globose, obtuse at apex, green and often mottled with white, often turning brown while still on plant, drying brown, moderately pubescent when young with gland-tipped stellate hairs like those of the stem but with rays often fused, these mixed with short simple glandular hairs, becoming glabrous to sparsely pubescent when mature. Seeds 25–50 per fruit, reniform, brown, rugose, 3–5 × 1.5–3.5 mm, the margin flattened with a swollen center.

**Figure 3.**
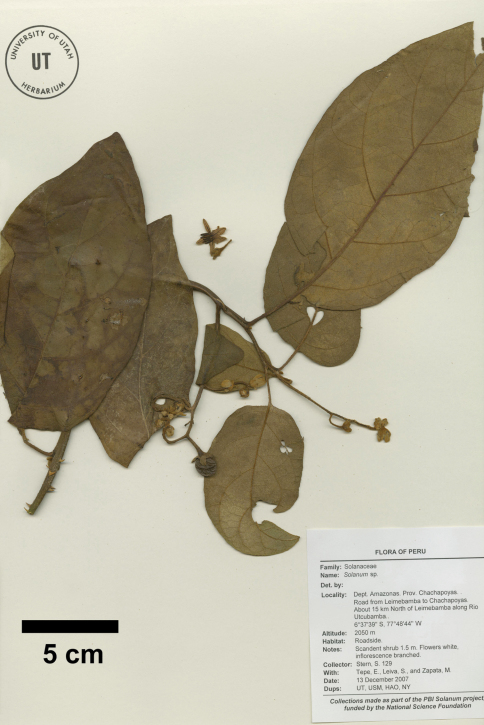
Isotype of Solanum achorum S. Stern [Stern et al. 129 (UT)].

**Figure 4.**
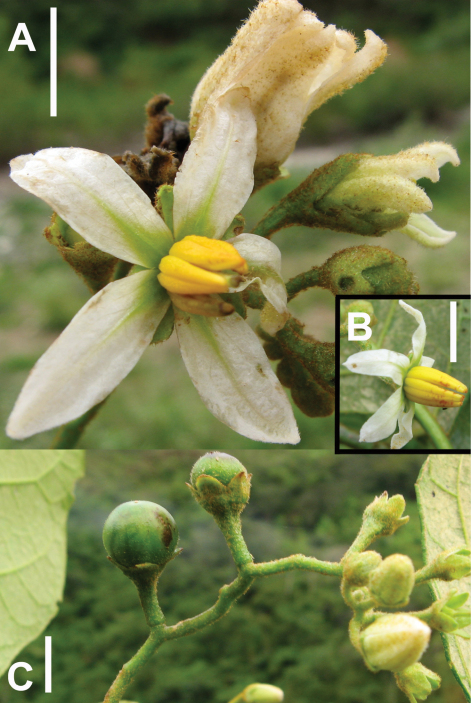
Photos of type collection of Solanum achorum S. Stern. **A** Hermaphroditic flower and buds. **B** Functionally male flower; note the absence of exserted style. **C** Inflorescence and immature fruits. Scale bars = 1 cm.

##### Distribution.

Known from northern Peru in Depts. Amazonas and Cajamarca and southern Ecuador in Prov. Zamora-Chinchipe in disturbed open places in montane tropical forest, 700–2100 m in elevation.

##### Ecology.

Flowering specimens were collected in December; fruiting specimens were collected in July, October, and December.

##### Etymology.

The name Solanum achorum is derived from the Greek “*achoros”* meaning “homeless.” This name was chosen because of disagreement as to which group within Solanum subg. Leptostemonum this species belongs.

##### Conservation status.

According to the IUCN Red List Categories (IUCN 2010) Solanum achorum is classified as VU- B2a+B2biii; D2 (Vulnerable). The extent of occupancy is estimated to be approximately 30,000 km2 with less than 10 collected locations. The conservation status of Solanum achorum is similar to that of Solanum rubicaule with respect to the potential of more unidentified specimens in herbaria, more specimens as a result of increased collecting efforts, and the difficulty of assessing future habitat as deforestation continues.

##### Specimens examined.

**Ecuador:** Zamora-Chinchipe: Cantón Chinchipe, Parroquía Zumba, Quebrada Tarrangamí, near cabin of Sandy León, W of Escuela Byron Jiménez, just S of Las Pircas, region of Guaramizal, 4°46'50"S, 79°12'33"W, 2000 m, 29 March 2005 (fr), L.Bohs et al. 3356 (LOJA, QCA, QCNE, UT); same locality, same date (fr), L.Bohs et al. 3358 (QCNE, UT); Cantón Valladolid, Parroquía Vallodolid, road between Valladolid and El Porvenir del Carmen, ca. 3 km from Valladolid en route to Tapala, 1600–1650 m, 4°33'27"S, 79°07'50"W, 1 April 2005 (fl), L.Bohs et al. 3380 (QCNE, UT); road between Zumba and Amaluza, 8–10 km W of Zumba, 1500–1700 m, 4°50'07"S, 79°09'50"W, 31 March 2005 (fl, fr), L.Bohs et al. 3367 (QCNE, LOJA, UT); along road between Zumba and Vilcabamba, 57.9 km N of Zumba, 9.2 km S of Santa Ana, 6.3 km N of Palanda, 4°36'39"S, 79°07'42"W, 1243 m, 28 July 2004 (fr), T.Croat 92480 (BM). **Peru:** Cajamarca: Prov. San Ignacio, Dist. San José de Lourdes, caserio Rumichina, limité con caserio Naranjos, 5°54'04"S, 78°36'09"W, 1811 m, 24 June 2006 (fr), J.Perea & V.Flores 2407 (BM); Prov. San Ignacio, Dist. San José de Lourdes, bosque alrededor de la comunidad, 5°06'16"S, 78°51'11"W, 1860 m, 10 October 2006 (fr), J.Perea & V.Flores 2799 (BM); Prov. San Ignacio, approximately km 115 on road from Jaen to San Ignacio, east side of hills dividing San Ignacio and Rio Chinchipe, 5°06'53"S, 78°59'16"W, 711 m, 17 December 2007 (fl, fr), S. Stern et al. 177 (BM, NY, USM, UT).

##### Discussion.

The plurifoliate sympodial units, ferruginous to reddish tomentum with stellate-glandular hairs, and large berries with large seeds and a pubescent exocarp identify Solanum achorum as a member of Solanum sect. Erythrotrichum. Additionally, parsimony analyses of sequence data from three molecular markers (nuclear ITS and *waxy* or GBSSI and chloroplast *trnT-F*) place Solanum achorum in this section; however, the relationships within the group are incompletely resolved due to a lack of taxon sampling and require further study (S. Stern and L. Bohs, unpub. data). The inflorescence structure of Solanum achorum, being branched with both hermaphroditic and staminate flowers, would place it in Agra’s (2008) subsect. Rhytidoandrum Agra; however, this relationship has not been tested phylogenetically using molecular data.

Of the 23 species of sect. Erythrotrichum that Agra (2008) recognized, only three occur in Peru, with one species, Solanum urubambaense Agra, endemic to southern Peru in the area around Cuzco. Solanum achorum can be distinguished from the two members of sect. Erythrotrichum occurring in northern Peru and southern Ecuador by a number of characters. It shares a similar vegetative appearance with Solanum megaspermum Agra, especially regarding habit, pubescence, and leaf shape; however, Solanum megaspermum has more robust inflorescences (> 30 flowers vs. 4–12 flowers in Solanum achorum) and larger seeds (5–5.5 × 2–3 mm vs. 3–5 × 1.5–3.5 mm in Solanum achorum). Solanum achorum also shares many characteristics with Solanum velutinum Dunal, including a scandent habit, similar pubescence, and similar-sized white corollas, but Solanum achorum has a branched inflorescence that can reach 20 cm versus inflorescences to 6 cm long in Solanum velutinum. The calyx lobes in Solanum achorum are 2–4 mm in length and not foliaceous while those of Solanum velutinum are commonly over 10 mm long and foliaceous.

## Supplementary Material

XML Treatment for Solanum rubicaule

XML Treatment for Solanum achorum
